# When and how is age mentioned? The role of age references in narratives of adults aged 65 and over

**DOI:** 10.1093/geront/gnaf276

**Published:** 2025-11-23

**Authors:** Veronika Gocieková, Anna Ševčíková, Gabriela Gore-Gorszewska, Martina Rašticová, Andrea Lambert South

**Affiliations:** Psychology Research Institute, Faculty of Social Studies, Masaryk University, Brno, South Moravian Region, Czech Republic; Psychology Research Institute, Faculty of Social Studies, Masaryk University, Brno, South Moravian Region, Czech Republic; Psychology Research Institute, Faculty of Social Studies, Masaryk University, Brno, South Moravian Region, Czech Republic; Department of Management, Faculty of Business and Economics, Mendel University, Brno, South Moravian Region, Czech Republic; School of Media and Communication, College of Informatics, Northern Kentucky University, Highland Heights, Kentucky, United States

**Keywords:** Beliefs, Stereotypes, Coping mechanisms, Well-being

## Abstract

**Background and Objectives:**

Both societal and personal beliefs about aging shape how older adults experience and respond to age-related changes. This study explores how adults aged 65 years and above internalize or resist aging stereotypes and beliefs through age-related references embedded in daily experiences.

**Research Design and Methods:**

Data were drawn from three research projects on relationships, sexuality, and employment in later life, each with a minimum age requirement of 50 years. For the secondary data analysis within this study, we selected 50 participants aged 65+ years from a total pool of 189 in-depth interviews. Thematic analysis methods were used to derive themes related to the context and function of age references.

**Results:**

Results highlight four key contexts in which participants referred to age: (1) *seeking balance between autonomy and limitations*, (2) *navigating societal versus personal expectations*, (3) *legitimizing end-of-life reflections*, and (4) *reframing hassles as unnecessary*. In these contexts, references to age served two primary functions: (1) *providing relief* and (2) *navigating decline*. (3) *Avoiding mentioning age* constituted a unique third function. Participants employed these references to manage experiences of decline and seek relief from the discomfort associated with aging.

**Discussion and Implications:**

Findings from the present study suggest that age references—including some rooted in ageist stereotypes—play a key role in how older adults navigate the aging process. These references help normalize limitations, express discomfort, or avoid confronting declines, thereby supporting adaptation and emotional balance. Recognizing their function may guide interventions that enhance adaptive uses while reducing risks of internalizing harmful stereotypes.

## Background and objectives

The aging process is accompanied by diverse societal beliefs and stereotypes that older adults may internalize and subsequently shape their self-perception, expectations regarding aging, and health behaviors (Levy, 2009). Prior research has shown that negative age-related beliefs can exacerbate stress and undermine well-being (Levy et al., 2016; Wurm et al., 2013). However, theoretical frameworks like the Strength and Vulnerability Integration (SAVI) model point to the existence of adaptive mechanisms, such as reappraisal, which help maintain emotional well-being despite age-related challenges (Charles, 2010). In this respect, the present study seeks to expand on this theory by exploring how older adults spontaneously refer to age and age-related beliefs in their narratives to understand the role of these references in dealing with age-related changes at older age.

### Aging and maintaining emotional well-being

Aging and the associated changes in daily functioning represent a potential chronic stressor (Smith & Baltes, 1998) that may challenge the biopsychosocial resources needed to adjust to age-related changes (Baltes & Baltes, 1993; Carstensen, 1992; Charles, 2010). Prior research and established theories such as the Selection, Optimization, and Compensation (SOC), the Socioemotional Selectivity Theory (SST), and the Strength and Vulnerability Integration (SAVI) model show that people are equipped with adaptive strategies that allow tackling age-related changes to preserve satisfaction, emotional well-being, and counterbalance losses (Baltes & Baltes, 1993; Carstensen et al., 1999; Charles, 2010).

For example, Baltes and Baltes (1993) explain that older adults can adapt to age-related changes by focusing on setting realistic goals and compensating for limitations, helping them maintain a high quality of life. The selection process can also play a role in social and emotional domains, fostering a positivity bias (Mikels et al., 2014; Reed et al., 2014). According to SST, older people become increasingly aware of the limited nature of their future, leading them to prioritize emotionally fulfilling experiences that maintain emotional well-being (Carstensen, 2021; Carstensen et al., 1999). In addition, the SAVI model incorporates insights from SOC and SST, suggesting that older adults use specific coping strategies, such as reappraisal and reframing, to preserve their emotional well-being later in life (Charles, 2010). These theoretical frameworks indicate that older adults can develop creative strategies to address age-related changes. These adaptive processes can also extend to using stereotypes or aging beliefs, which may help individuals navigate challenges by aligning their aging expectations with societal norms. Maladaptive aging beliefs and ageist stereotypes can be reframed by older adults in a more optimistic light, allowing them to view these perceptions as potentially beneficial rather than detrimental. However, little is known about the reframing process, including how individuals apply their age-related beliefs and references in everyday life to navigate the challenges of aging.

### Aging and age-related beliefs

Throughout life, individuals are continually exposed to various aging beliefs and stereotypes. These societal norms often intersect with personal beliefs and experiences (Cary & Chasteen, 2015; Hummert, 2011). Personal beliefs about aging that reflect individual perceptions are closely connected to stereotypes representing broader societal views. Older adults may internalize age-related stereotypes, particularly when they perceive them as relevant and identify with the age group (Cary & Chasteen, 2015; Chasteen et al., 2002; Hess et al., 2003; Levy, 2009). Such notions about aging often operate unconsciously, becoming prominent in the process of self-definition and subsequently influencing expectations and behaviors (Levy, 2009).

It is essential to acknowledge the substantial evidence linking negative age-related beliefs and stereotypes to experiencing stress (Levy et al., 2016; Quach, 2020; Robertson, 2016). These age-related stressors can adversely affect health behaviors and well-being (Levy, 2009; Wurm et al., 2013). Ageist beliefs and stereotypes often foster the perception that modifying health behaviors in response to age-related issues is futile, resulting in lower compliance among older patients. However, Wettstein and his colleagues (2021) found that the negative association between perceived stress and negative attitudes toward one’s own aging diminishes with age, suggesting that the role of age-related references might evolve.

Some evidence suggests that older adults may consciously and unconsciously activate age-related beliefs to alleviate stress in challenging situations. These mechanisms have been observed particularly in the context of later-life sexuality, where invoking age was associated with relief when managing difficulties. For example, older adults experiencing unsatisfactory sexual experiences due to diminished sexual function may come to view sexuality as less relevant or appropriate for their age, leading them to disengage from this aspect of their lives (Gore-Gorszewska, 2023; Ševčíková & Sedláková, 2020). Importantly, many individuals find this perspective calming and relieving. Another study found that the more age-related sexual stereotypes people held, the less importance of sex they reported. However, these stereotypes appeared to buffer the negative impact of sexual difficulties on the importance placed on sex among people aged 50 and older (Gocieková et al., 2024). Similarly, in the work domain, adopting a more passive stance, such as “standing back” or redefining oneself as “semiretired,” has been shown to reduce or regulate negative emotions, which may stem both from experiences of age-based discrimination and from actual declines in performance (Grima, 2011; Harris et al., 2018; Levy, 2017). Staudinger et al. (1999) also identified a potential protective effect of “giving up” in situations where few viable alternatives exist, suggesting that accepting certain beliefs may help to reduce stress and societal pressure. For instance, older adults may adopt societal beliefs such as “older people are not tech-savvy,” enabling them to avoid the strain and risk of failure associated with keeping up with rapid technological advancements. This adaptation eases emotional stress and highlights the complex role of age-related beliefs in later life. These fragmentary findings suggest that such beliefs can serve as an important coping mechanism across various aspects of life.

### Age-related references

While limited research focuses on how older adults use age-related references in their narratives, existing qualitative studies provide valuable insights into how older adults mention age in various contexts. Age mentions often arise when individuals seek to resist or distance themselves from aging and its implications (Buckley et al., 2015; Clarke & Warren, 2006; Okun & Ayalon, 2023; Rizzolino, 2021). Age is also frequently referenced in relation to health and life changes or in reflections on mortality (Buckley et al., 2015). For example, individuals normalize these experiences by attributing them to aging (“It’s just because I’m getting older”) or, conversely, express feelings of helplessness and frustration about their situation (“My mind wants to do these things, and my body’s saying you can’t”) (Buckley et al., 2015; Reichstadt et al., 2010; Rizzolino, 2021). When asked about successful, happy, or healthy aging, individuals often mention acceptance of aging and contentment with their current life circumstances (Mantovani et al., 2016; Reichstadt et al., 2010). One participant noted that this acceptance brought her a sense of peace and life satisfaction (Reichstadt et al., 2010).

Through these references, older adults may also articulate broader beliefs and stereotypes about what aging entails, such as assumptions about physical decline or social roles. These beliefs can help normalize the challenges of aging, fostering a sense of authenticity and life satisfaction. Findings to date suggest that age-related references promote well-being by helping individuals navigate their perceptions of aging and integrate them into a meaningful narrative.

### Current study

This study examines the potential for age-related beliefs to function adaptively in various aspects of life, especially in light of the diminishing biopsychosocial resources accompanying aging. By exploring age-related references in the narratives of older adults, we aim to gain insight into how age-related references and beliefs are woven into daily experiences. Understanding the circumstances in which individuals mention age offers valuable perspectives on how these beliefs shape identity, influence decision-making, and impact overall quality of life. In this respect, secondary analysis was chosen as an analytical approach as it allows observations of how age references spontaneously emerge without respondents being specifically prompted. The decision to focus on adults aged 65 years and above aligns with the classification used by the Czech Statistical Office (ÚŘAD Č.S 2020), which designates individuals aged 65 and older as *seniors*. This classification coincides with often-negative societal assumptions about aging, including that social relationships and health will inevitably decline after 65, and that retirement from paid work is socially accepted and often viewed as desirable. Thus, the following research questions (RQs) are posed:

RQ1: In what contexts do age references appear in the narratives of adults aged 65+?

RQ2: What functions do age references serve in the narratives of adults aged 65+?

## Research design and methods

### Participants

This study draws on the secondary analysis of data from three distinct research projects conducted in Czechia with focus on exploring specifics of later-life sexuality (*n *= 30; age range = 50–75 years; *M* (age) = 61.1; *SD *= 6.76; *Mdn *= 63), partnerships (*n *= 31; age range = 61–81 years; *M *= 72; *SD *= 5.11; *Mdn *= 72), and employment in three sectors: nursing, public transportation, and financial institutions (*n *= 128; age range = 50–78 years; *M *= 56.7; *SD *= 5.8; *Mdn *= 56). The datasets were available to the authors under a collaboration agreement. Each dataset comprised a unique set of participants, with no individuals appearing in more than one project. Data were collected between 2017 and 2021 using similar recruitment strategies (a convenience sampling method and a snowball technique) and employing in-depth or semi-structured interviews that were recorded and transcribed while removing all identification information. Detailed descriptions of the recruitment strategies and the scope of each project are provided in studies by Ševčíková and Sedláková (2020), Rabelová et al. (2024), Dudová (2021), and Křížková et al. (2019). The projects were approved by the Research Ethics Committee of Masaryk University (EKV-2016-035, EKV-2019-023) and data collection was conducted per the institutional ethical guidelines.

These three datasets were chosen to represent key aspects of life—relationships, work, and sexuality—that undergo significant changes in later life. Together, they offer a comprehensive view of the challenges and adaptations associated with aging. Using multiple datasets also broadens the exploration of how age references and age-related beliefs spontaneously arise in different contexts, offering more profound insights into the diverse experiences of older adults.

In total, 189 interviews were conducted across the three research projects. For this study, we focused only on participants in late adulthood, so we selected all interviews (*n *= 50) with individuals who met the age criterion of 65 years and above. The final sample included participants aged from 65 to 81 years (*M *= 71.04; *SD *= 4.76; *Mdn *= 69), with women being slightly overrepresented (58%). In the datasets on later-life sexuality and partnerships, all participants aged 65 and above identified as heterosexual, except for one who self-identified as gay. All informants in the final sample lived independently and self-reported no diagnosis of significant cognitive impairments. Further details of their demographic characteristics are described in Table 1. It is important to note that the study was conducted in Czechia, which is an ethnically and racially homogeneous country, with all participants identified as White European. To ensure participant anonymity in this secondary analysis, pseudonyms were assigned to all respondents.

**Table 1.** Characteristics of participants aged 65 and over.

Characteristics	Number of respondents within datasets on			Total sample
	Sexuality	Relationships	Employment	
**Age**				
** 65+**	10	29	11	50
**Sex**				
** Female**	7	18	4	29
** Male**	3	11	7	21
**Education**				
** Primary**	1	0	1	2
** Secondary**	6	16	6	28
** Higher**	3	13	4	20
**Occupation Status**				
** Working**	1	4	2	7
** Self-employed**	0	2	0	2
** Part time**	2	2	5	9
** Retired**	7	21	4	32

### Data analysis

Secondary data analysis explored when age references emerged and with which function. This approach allowed the examination of spontaneous mentions of age and their function, providing insights that might be missed in studies where specific or potentially leading questions are posed. By drawing from various datasets, we could observe a broader spectrum of age-related mentions across diverse topics, gaining a more comprehensive understanding of when and how age becomes relevant to older adults. The secondary analysis was conducted using thematic analysis, which is well suited for the nature of the data and research questions (Braun & Clarke, 2006). Using this flexible method, we were able to identify and provide a rich description of patterns in secondary data. Two main themes—*context* and *function of age references*—were predefined as focal points for the analysis.

In accordance with Braun and Clarke’s (2006) six-phase approach, a bottom-up method was adopted. A bottom-up approach includes (1) familiarization with the data, completed by the first author, who repeatedly read through the interviews, highlighted relevant sections, and took notes. Subsequently, (2) initial codes were generated (e.g., accommodating to the slower phase with aging, explaining work changes through age), and meaningful units were created and described, often including quotes from respondents. Further steps (3) involved searching for themes *and* the codes were consolidated and compared across interviews. At this stage, regular discussions with the coders took place to ensure the accuracy and depth of the identified themes. (4) Themes were then reviewed and adjusted with coders to ensure consistency across the dataset. The process of (5) defining and naming the themes was refined through further discussions with the coders, with some themes merged into larger, overarching themes as they were found to address the same central ideas or patterns (e.g., “health is good for my age”, “independence and care”, and “changes in activities and employment” were merged to “seeking balance between autonomy and limitations in later life”). Throughout the process, notes were taken to facilitate (6) the final report, ensuring that key findings were adequately presented. The first author translated the quotes and subsequently checked for accuracy with the second author. The quotes presented in the “Results” section were selected to illustrate the identified themes most effectively, but they represent patterns observed across the entire dataset.

Age references appeared relatively evenly across all three datasets. However, their specific content reflected the focus of each project (e.g., interviews on sexuality included more age references related to sexual activity). While the analysts primarily relied on spontaneous narratives, in all three datasets, participants were asked how aging interferes with the studied aspect of their life. In the analysis, we carefully distinguished between spontaneous mentions and responses prompted by such questions, ensuring that no artificial connections to age were inferred. Interestingly, the absence of any reference to age even in response to direct questions emerged as a relevant analytical category, which we describe in the results as a tendency to avoid acknowledging age.

## Results

**Figure 1.**
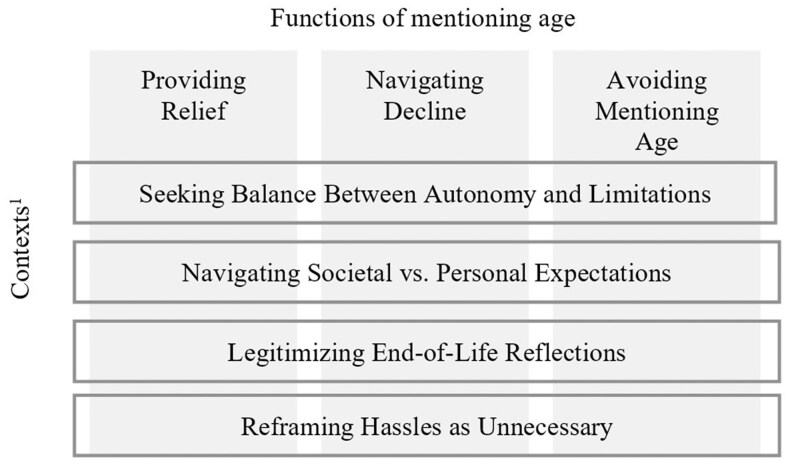
Functions of mentioning age. Alt text: Figure showing three functions of mentioning age—providing relief, navigating decline, and avoiding mentioning age—displayed as vertical columns. These functions are linked to four contexts shown horizontally: seeking balance between autonomy and limitations, navigating societal versus personal expectations, legitimizing end-of-life reflections, and reframing hassles as unnecessary. A footnote states that contexts refer to situations where the functions of mentioning age were observed.

This study examined how participants referenced age across various life domains and revealed a complex interplay of overlapping functions and contexts. We identified four primary contexts in which participants mentioned age: (1) *seeking balance between autonomy and limitations*, (2) *navigating societal versus personal expectations*, (3) *legitimizing end-of-life reflections*, and (4) *reframing hassles as unnecessary.* Each of these contexts intersected with one or more functions that age references had in the narratives, namely, *providing relief* and *navigating decline*. We also identified the pattern of *avoiding mentioning age* with a unique role, as some interviewees refrained from bringing up age even in contexts where it appeared relevant or could have been an obvious explanation (Figure 1).

### Contexts of age references

#### Seeking balance between autonomy and limitations

Participants frequently mentioned their age in the context of perceived autonomy and freedom. They highlighted the advantages of retirement, such as fewer obligations and the possibility to choose from various options, which enabled them to work part-time or select (work) tasks that they enjoyed. For instance, a 65-year-old man, Pavel, noted,

It’s interesting, but as I got older, I started to enjoy my work more because I had this wonderful perspective that I can work when I want and do what I want. Now we have a so-called service exchange on the computer, where the manager posts what’s not covered, and I can choose [a task] I like. (65M_int49)

Some participants viewed retirement as a time to prioritize time spent with loved ones over responsibilities. For example, Julie and her husband chose to give up working at the swimming pool as they wanted to make the most of their retirement and had sufficient income from their pensions: “Now we are together, and because we are old enough and want to enjoy our retirement, we gave up the swimming pool. I’m still with the footballers, at the ticket office” (Julie, 74 W_int22). This decision exemplifies a balance between financial security and the freedom to choose activities that bring fulfillment and enjoyment, such as time spent together. For Julie and her husband, this choice reflects an autonomy gained through retirement, where they feel free to embrace activities of personal significance.

However, participants also acknowledged the challenges of maintaining autonomy in later life. Age was mentioned to recognize growing physical and cognitive limits that have to be balanced, for instance, by lowering pace and increasing caution: “Even if you know the highway to the city well, you have to be more mindful. You will see when you’re older and drive a car, you can’t drive automatically” (Jan, 78M_int40). This may illustrate cases when advancing age may signal a necessary shift in how individuals engage with familiar routines because daily tasks begin to require greater attention.

Anna, a woman in her 70s, described her profound realization of aging in these words: “After 70, you suddenly feel old… I started to realize that I couldn’t keep up with many things. In the garden, I’d get tired, or we went cycling, and I couldn’t manage what I used to” (Anna, 75 W_int19). The way Anna framed her narrative indicated her growing acceptance of physical limitations but also the challenging emotional adjustments required to cope with reduced capabilities.

Age and autonomy were also discussed in the context of caregiving, where the fear of becoming a burden became prominent. One interviewee reflected on the potential loss of independence:

I’m aware that a lot of people don’t even live to this age… What’s the point of living to 85 if I spend five years being a burden… like a demented person? It’s not acceptable to me; one should die on time, but how do you make it work? (Karel, 75M_int1)

This reflection underscores the tension between valuing longevity and fearing a loss of autonomy. It shows how some participants grappled with maintaining dignity and control over their lives, even in the face of an inevitable decline. These insights highlight that autonomy is about physical independence and retaining the ability to make personal choices, even as aging introduces new constraints. By focusing on self-direction, participants framed autonomy as essential for preserving dignity and a sense of control in later life.

#### Navigating societal versus personal expectations

Participants spontaneously mentioned age in relation to societal expectations and norms internalized over the life course. Specifically, some interviewees conveyed discomfort when they failed to meet personal or societal expectations of aging. For example, 69-year-old Iva expressed shame for not reaching the level of independence and assertiveness she had expected in later life. She had anticipated being more autonomous and capable of standing up to her husband but felt disappointed that this shift had not occurred:

He lives his own life, decides what he will do, and it’s more me who asks about everything, asks for his permission. I’m almost ashamed of it, that I’m so old, but that’s just how it has come to be, and that’s how it is, that I somehow foolishly submit to him. (69W_int32)

In some cases, societal expectations did not align with participants’ personal expectations and views. This was particularly visible in the area of sexuality. Some participants perceived sexual expression to be less relevant or not appropriate in old age, while they witness societal pressure to maintain sexuality in later life: “I think it’s normal for our age (not to have sex), but nowadays there is pressure to maintain sexual activity even into old age” (Dana, 80 W_int13). In Dana’s account, age was mentioned in the context of the clash between old and new societal expectations about how people should age and what should be part of aging or successful aging (e.g., not interrupting lifelong activity such as partnered sex). Dana did not adhere to the societal pressure but acknowledged its impact. Other participants were not so adamant and admitted to adhering to societal expectations at the expense of their own views on how one should behave in older age.

#### Legitimizing end-of-life reflections

References to age frequently gave participants an opportunity to express their thoughts on the end of life. Reflections on mortality often became more pronounced as they grew older, leading to a greater willingness to discuss topics such as death, wills, and funeral plans. Age provided a socially acceptable entry point into these conversations, framing them as sensible and necessary. For example, 74-year-old Adam described how he and his wife regularly discuss their final wishes: “We’re getting to the age where we can talk about this topic.” He noted that once infrequent conversations had become routine, driven by the awareness of their advancing age. His mention of life expectancy statistics and his age helped him rationalize the proximity of death: “and I’m 74 years old, so to accept the idea that I might pass away in a few years… it can either scare me or I can accept it. Nobody’s staying here…” (74M_int24).

Adam’s thoughts illustrate how some participants use their advancing age as a means to approach sensitive subjects with less emotional weight, helping them normalize the inevitability of death. This acceptance offered relief for Adam, allowing him to feel more in control of his end-of-life decisions and less burdened by fear.

In addition to these solemn reflections, participants often employed humor to hint at their aging and the approaching end of life. Alena, a woman aged 70, joked, “Who will go first up the hill [a euphemism for a graveyard]?” (Alena, 70 W_int29). At the same time, another participant, Adam, lightheartedly remarked, “Every night we say to each other, ‘see you in the morning?’ and she [his wife] replies, ‘God willing.’ Influenced by my teachers, I say ‘inshallah’” (Adam, 74M_int24), humorously acknowledging that whether they wake up the next morning is not guaranteed and in the hands of a higher power. These humorous remarks allowed participants to process the inevitability of death in a lighthearted and communal manner, offering comfort through shared experiences. By expressing thoughts about aging and death in this way, interviewees seemed to find relief and a sense of solidarity, as they were able to openly discuss what is often left unsaid, reinforcing their acceptance of the journey they navigate together.

#### Reframing hassles as unnecessary

As individuals age, they often experience a complex interplay between the desire for stability and the necessity of adapting to change. Many interviewees gradually legitimized the idea that certain aspirations, activities, or lifestyle changes are no longer necessary, reinforcing their preference for stability and familiar routines. For example, Pavel’s aversion to moving was framed in his narrative as a practical decision, acknowledging the risks and stress that such a change could bring at an advanced age. His reluctance to face the uncertainty of future relocations underscores the sentiment that, at a certain point, some things—like moving or making major life changes—are no longer worth the trouble: “These matters are, in my opinion, unnecessary” (65M_int49).

Moreover, participants’ notions of aging not only served to legitimize the aging process itself but also allowed them to find an acceptable position within it. This was evident in 68-year-old Jakub’s reflection on his changing desires and priorities:

I can’t walk as much these days… actually, I don’t really want to travel much anymore. As one ages, they start seeking happiness in things around them, in the closest things. I’m returning to the cottage where I used to be happy, studying flowers, grass, smaller areas… I always wanted to know what was beyond the horizon, what was beyond that mountain ridge, but today… first, I already know, and second, I don’t really feel like overcoming that anymore. (68M_int35)

Here, the shift in focus from ambitious goals to valuing smaller, more accessible pleasures illustrates an important aspect of this adjustment process. By rationalizing the change in their desires, participants like Jakub position this transition not as a loss but as a legitimate adjustment to their stage in life. In this way, they frame their reduced aspirations as a source of wisdom and contentment rather than deprivation. This redefinition of goals helps participants embrace aging, accepting the changes it brings while maintaining a sense of fulfillment.

### Function of age references

The analysis of participants’ narratives on aging led to identifying three key functions that age references serve in their lives: providing relief, navigating decline, and avoidance of mentioning age. These functions did not appear uniformly. In some narratives, only one function surfaced within a single context; in others, all three functions emerged across multiple contexts. Each participant’s narrative varied, with some utilizing a single function or context while others demonstrated a blend of multiple functions in different contexts. For instance, a participant might discuss *navigating decline* within the context of *legitimizing end-of-life reflections*, while later, *providing relief* might emerge within *seeking balance between autonomy and limitations*. These varied overlaps highlight how participants uniquely engaged with age-related themes, with each conversation reflecting a distinct approach to the personal and contextual relevance of aging.

#### Providing relief

In discussing their experiences, participants often referenced age to cope with and normalize the adjustments encountered in later life. This approach provided relief from the pressures of maintaining past lifestyles, which, for many, were no longer feasible. These findings shed light on how embracing these beliefs can function as an effective coping mechanism.

In *seeking balance between autonomy and limitations*, participants frequently used humor to address age-related health concerns, such as physical limitations or memory lapses. For instance, 75-year-old Jana described moments of shared forgetfulness with her husband:

(As we age), we might both forget something and want to talk about it. We look at each other and start laughing because we both know what it is, but we just can’t say it. We’re both… a bit dim-witted (laughs)… Really, it’s funny how stupid we are (laughs). (75W_int20)

In this way, referencing age allowed Jana and her husband to acknowledge limitations while reinforcing a sense of shared understanding, contributing to emotional relief and acceptance of declining abilities.

When *navigating societal versus personal expectations*, age-related references also provided participants with a framework for countering societal pressures that conflict with their values or comfort levels. For example, Dana (80 W_int13) expressed feeling societal pressure to maintain sexual activity in old age, as noted in an earlier example. She decided it was acceptable not to follow societal expectations, choosing instead to value her perspective on aging. By validating her preference against societal norms, she relieved herself from the stress of expectations that felt irrelevant to her stage of life.

In *legitimizing end-of-life reflections*, age references helped participants contextualize health issues or mortality in ways that may reduce anxiety. In addition to the account of Adam, presented above, also 72-year-old Sara acknowledged the inevitability of health challenges at the end of life with humor: “It’s true that at this age, you might have more health concerns… but you just take it in stride because when you look at your birth year, you realize it has to be this way (laughs)” (72 W_int26). By framing her health issues as an expected state in later life, Sara used age to legitimize her experience, turning an otherwise difficult topic into something more manageable and even humorous.

Finally, in reframing *hassles as unnecessary*, some participants found relief in adapting their behaviors to align with their current capacities and priorities. For example, as previously mentioned, 68-year-old Jakub reflected on his changing desires, describing how he shifted his focus from ambitious goals, like traveling and exploring new horizons, to appreciating smaller, more accessible pleasures in his immediate neighborhood. This adjustment allowed him to embrace the realities of aging by prioritizing contentment versus overexertion.

Jakub’s perspective illustrates how embracing adaptations to life’s current stage can transform potential limitations into meaningful opportunities for well-being. Here, age functioned as a justification for redefining priorities without guilt, enabling participants to align their goals with their present needs and capacities.

In these varied contexts, participants’ references to age served as a way to navigate aging with a sense of relief and self-compassion, enabling them to reinterpret limitations as natural adjustments rather than losses. This acknowledgment provided a foundation for continued life satisfaction in later years.

#### Navigating decline

Participants frequently referenced their age to articulate and process various losses they were experiencing. These age-related mentions often served as markers for expressing dissatisfaction, frustration, or a sense of resignation toward the inevitable declines that accompany aging, providing means to confront and make sense of these changes.

In *seeking balance between autonomy and limitations*, many participants lamented the gradual loss of physical strength and energy, framing it as a natural but disappointing aspect of growing older. For instance, 81-year-old Lenka remarked, “the process of aging… it’s a shame, you know, we no longer have the energy to tackle anything” (81 W_int11). Here, referencing age allowed her to express her frustration with the physical limitations that increasingly shaped her daily life.

Similarly, in *navigating societal versus personal expectations*, participants occasionally invoked age to address their frustration with societal norms and their own needs. When discussing her relationship situation, currently single Nina articulated her frustration with societal perceptions of desirability, stating, “nobody would want an old granny, would they” (66 W_int38). It was visible in her account that Nina struggled to accommodate her personal view that having a younger partner would better match her energy levels with the societal notion that women her age are often deemed less desirable. She conveyed resignation about the impossibility of establishing the kind of relationship she would have imagined for herself.

In *legitimizing end-of-life reflections*, participants used age references to process mortality and social losses that accompany aging. Jana shared her experience with shrinking social circles:

We are among the few who are left, we don’t have any acquaintances anymore. There used to be many couples; we used to meet, go to the pub together, go on vacations, and now we are the only ones left after so many years. (75W_int20)

In this case, age served to understand and contextualize losses as a natural part of life’s course, giving voice to Jana’s feelings of isolation while validating them as an expected outcome of aging.

Last, within *reframing hassles as unnecessary*, some participants used age to frame interpersonal challenges that had become more common with age but were now too draining to address. Dan shared his frustration about how his wife’s increasing emotional sensitivity occasionally led to conflicts, which he called “small storms”: “… as (wife) gets a bit older, she’s more sensitive than she used to be, and that’s upsetting” (74M_int21). While he expressed dissatisfaction with this change, he also indicated it was no longer worth arguing about it, viewing it as a frustrating but unchangeable aspect of aging that he had reluctantly come to accept.

In these instances, age was used as a framework to express and rationalize, in some cases, the inevitable physical, emotional, or social losses inherent to aging. It gave some participants an outlet to voice various negative emotions, such as sorrow, resignation, or frustration. It also allowed them to accept changes they see as unwelcome yet inevitable.

#### Avoiding mentioning age

A specific phenomenon was observed in some of the interviews where participants did not perceive themselves as old. In cases where age or aging seemed particularly relevant but was not mentioned by participants themselves, interviewers occasionally asked directly whether and how the discussed topic related to aging. These follow-up prompts often resulted in evasive answers or rejections of the notion that aging had any impact on their lives. Alternatively, some participants referred to older individuals as an outgroup: “Those old people were right…” (Anna, 75 W_int19). This tendency to avoid acknowledging age appeared across all four identified contexts, illustrating how participants navigated aging differently based on the setting and interaction. Notably, for some interviewees, this avoidance was present in all contexts, while for others, it surfaced selectively, marking their narratives only within particular contexts.

In some cases, this phenomenon appeared in the form of discrepancies in how persons behaved and were perceived by their significant others, such as spouses. Kira described this dynamic: “he (my husband) still thinks that… he’s a hero (laughs), that… he can do everything [at his age], but it’s not possible. And thus, conflicts arise” (69 W_int31)

Within couples, this discrepancy regarding the perception of aging occasionally created points of friction, as in Kira’s example. However, the denial of age and the aging process functioned more often as a way of coping with the changes that come with aging. This approach frequently emerged in the domain of sexuality, where respondents felt satisfied with their intimate relationships when sex remained a part of their lives, thereby avoiding or negating the impact of aging. Participants emphasized that maintaining partnered intimacy, leading to a sense of youth, contributed to their well-being:

I know that someone… might say, “You’re old, what are you doing?” and such… My husband has never said that to me, ever. On the contrary, when I’m intimate with him, I still feel young. And that is really nice, you know? (Sofie, 65W_int8)

This statement indicates that ignoring aging and associated changes can serve as a source of support. The flexibility with which participants applied this strategy—either across all contexts or selectively in certain areas—underscores how denying or reframing age could alleviate perceived threats posed by the acknowledgment of decline.

## Discussion and implications

This secondary qualitative analysis of three datasets explored the context and the function of age references that spontaneously emerged in the narratives of adults aged 65 and older. The study found that these references may offer unique forms of relief and help navigate experiences of decline in ways that have not been extensively highlighted in previous research.

The findings on relief align with existing literature, which emphasizes the critical role of acceptance in promoting subjective well-being during later-life transitions. Dockendorff (2014) highlights that subjective well-being can remain stable—or even improve—despite losses, provided individuals adjust their goals and preferences to changing circumstances. Importantly, this acceptance must avoid fostering feelings of helplessness, isolation, or resignation, as these are identified in the literature as significant risks (Dockendorff, 2014; Fuller & Huseth-Zosel, 2021).

Our study seems to reveal a significant nuance here: Participants adjusted their goals and actively leveraged age-related beliefs to reappraise their stressful situations. For instance, framing certain losses as “normal for this age” allowed participants to reframe experiences of decline as part of a broader life narrative, effectively mitigating emotional distress associated with feelings of personal failure. This may suggest a previously underexplored adaptive dimension of age-related beliefs that supports resilience. Our findings indicate that some age-related beliefs (e.g., sex as no longer part of later life) may be reframed by individuals in a positive light, thus supporting adaptive strategies like the positivity bias. In this context, the present study extends the SAVI model by proposing that some age-related beliefs and stereotypes may act as reappraisal tools, aiding healthy adaptation and enhancing subjective well-being, something that has not been previously emphasized in existing research (Gocieková et al., 2024; Gott & Hinchliff, 2003; Levy, 2009; Ševčíková & Sedláková, 2020). Notably, this reframing does not uniformly suggest passive acceptance but often reflects a strategic, deliberate process of self-preservation.

A new finding seems to highlight the tension between societal expectations and individuals’ perspectives on aging. Concepts such as “healthy,” “successful,” or “active” aging are often vaguely defined when commonly used, which can create undue pressure on older adults, especially when their lived experiences do not align with societal ideals. This societal narrative may foster unrealistic or harmful expectations because it frequently overlooks the physical changes that naturally accompany aging (Lamb, 2014; Reich et al., 2020). Some participants’ interactions within their social environments underscored this disparity, emphasizing the disconnect between their personal beliefs and externally imposed standards (Rizzolino, 2021). While older adults may possess unique and meaningful views of aging, these perspectives often diverge from conventional societal narratives of successful aging. This highlights the need for a careful redefinition of such ideals, an aspect that the present study emphasizes more directly than previous research. However, the narratives in this study suggest that older adults can exhibit a critical awareness of these societal pressures, selectively internalizing or resisting them based on their values. For example, one participant rejected the societal expectation of sexual activity in later life, framing this choice as consistent with her personal views, thereby reaffirming her autonomy. This dynamic reinforces the importance of validating diverse pathways to aging well.

A notable context in which age references appeared and had a positive role was the narratives about the end of life. While this topic is not new, age references facilitate its articulation, allowing participants to openly engage with the realities of life’s final stages, which have not been extensively documented. Such openness aligns with existing research on the importance of addressing end-of-life values, preferences, and dialogue to empower older adults and improve the quality of care (Towsley et al., 2015; Yip et al., 2024). Avoidance of these conversations can hinder necessary discussions and planning, a point underscored in the present study, and also shown in Rizzolino (2021). In addition, the types of humor used in Czech participants’ end-of-life narratives corroborates U.S. research, which found similar humor types when discussing end-of-life topics (Lambert South et al., 2022).

Despite the extensive sample, spontaneous mentions of age were relatively sparse, with some of the participants’ narratives suggesting patterns of avoidance or explicit rejection of age-related references. This avoidance often seemed to function as a source of support and a way to maintain self-esteem, with denial or reframing of age appearing to serve to alleviate the perceived threats associated with acknowledging decline. Dubovská et al. (2017) also showed the frequent occurrence of age denial in Czechs with the function of self-protection from negative thoughts associated with aging. This view is in line with findings by Rizzolino (2021) and Okun & Ayalon (2023), who highlight its protective role. Avoidance or explicit rejection of age-related references may also represent a response by older individuals to the negative and stereotypical labels often associated with aging, which portray the older adults as useless or obsolete. According to Kuypers and Bengtson (1973) older people frequently experience role loss, ambiguous norms, and a reduced number of social reference groups, resulting in a lack of meaningful feedback regarding their identity and value. These factors may increase older adults’ susceptibility to negative societal labeling and psychological decline. In this context, older individuals might disengage from restrictive social judgments by avoiding age-related references.

Our findings indicate that acceptance and accommodation in aging do not necessarily equate with “giving up.” Rather, they represent a process of adjustment that offers relief, emotional well-being, and opportunities for continued self-discovery. Participants in this study found comfort in reframing age-related challenges and embracing the natural course of aging, allowing them to maintain a sense of purpose and fulfillment. This highlights the importance of exploring the role of age-related beliefs and stereotypes when working with older adults. By recognizing the dual role of age references—as both tools for adaptive coping and potential sources of stress—it becomes possible to design interventions that reinforce their positive aspects while mitigating the risks of internalizing harmful stereotypes. For example, promoting narratives that inform about resilience and adaptation could counterbalance societal narratives that equate aging with decline.

These insights have significant implications for practitioners, policymakers, and caregivers, suggesting that efforts to improve older adults’ well-being should include strategies to validate diverse aging experiences and support autonomy in defining what successful aging means on an individual level.

## Limitations and future research

A limitation of this study is its focus solely on the Czech population. Future research could explore the extent to which findings of this study can be applied to other cultural contexts and whether age-related references and beliefs are employed and resisted in comparable ways. Such cross-cultural comparisons would help determine whether the patterns identified here reflect broader tendencies in aging narratives or whether specific nuances arise due to differences in cultural, historical, or socioeconomic contexts. For instance, the advancement of aging policies and cultural expectations of later life may shape the extent of avoiding mentioning age. A notable aspect of our findings is that cognitive decline was infrequently mentioned. While participants occasionally referred to growing physical and cognitive limits, they did not elaborate on more profound changes in cognitive functioning. This could be related to the characteristics of our sample, as none of the participants reported being diagnosed with significant cognitive impairments. Another possible explanation is that one-fifth of our sample came from a dataset focused on employment, including individuals still working in sectors with high responsibility, where admitting to cognitive decline could potentially conflict with their professional standing and job security.

Future research, especially large-scale studies, should assess the extent to which age-related references are employed to mitigate emotional distress associated with feelings of personal failure, and how this tendency varies depending on gender, age, health, and other key factors that may moderate the adoption of ageist beliefs. Intersectional perspectives (e.g., considering race or ethnicity in other national contexts) may reveal additional layers of meaning or highlight structural inequalities that shape aging experiences.

However, this limitation was mitigated by the use of three distinct datasets covering significant areas of life, which allowed for a more comprehensive and nuanced understanding of participants’ perspectives. Finally, while the findings provide rich insights, exploring the views of others in the older person’s life (e.g., spouses, adult children) could add further texture and depth to the data. Future research should integrate complementary methods, such as longitudinal or observational designs, to further validate and expand on these results.

## Conclusion

This study revealed that age references in older adults’ narratives serve diverse functions, including legitimizing aging, coping with change, and navigating societal expectations. While some highlighted tensions between lived experiences and societal ideals of “successful” aging, many references provided individuals with relief and self-acceptance, even though they sometimes drew on ageist stereotypes of unavoidable losses and decline. These findings underscore the complex role of age-related personal beliefs in shaping experiences of aging and their potential to support emotional well-being when framed adaptively. If upheld in future research, these insights may challenge common, negative, and pessimistic understandings of adjustment to aging, suggesting instead a dynamic process of acceptance and self-discovery that holds promise for enhancing well-being in later life.

## Funding

This work was supported by Masaryk University [grant number MUNI/A/1736/2024] and is part of the project “Research of Excellence on Digital Technologies and Wellbeing” (CZ.02.01.01/00/22_008/0004583), co-financed by the European Union.

## Data Availability

This study used secondary qualitative data, and the research team does not own the original datasets. Due to legal and ethical agreements established during the initial data collection, the de-identified data cannot be shared directly by the authors. However, the datasets are accessible through their primary sources. Specifically, datasets on later-life sexuality and partnerships are available upon request by contacting the first author of this paper, and the dataset on employment is available upon request from the fourth author. The study was not preregistered, as preregistration was not required for the qualitative research design or analytic plan.
